# Revised methane emissions factors and spatially distributed annual carbon fluxes for global livestock

**DOI:** 10.1186/s13021-017-0084-y

**Published:** 2017-09-29

**Authors:** Julie Wolf, Ghassem R. Asrar, Tristram O. West

**Affiliations:** 1 USDA-ARS, Adaptive Cropping Systems Laboratory, 10300 Baltimore Ave., Building 001, Room. 342, BARC-WEST, Beltsville, MD 20705 USA; 2 Joint Global Change Research Institute, 5825 University Research Court, Suite 3500, College Park, MD 20740 USA; 3 US Department of Energy, SC-23, 1000 Independence Ave., Washington, DC 20585 USA

**Keywords:** Methane emissions, Carbon monitoring system, Livestock, Enteric fermentation, Manure management, Greenhouse gas, Carbon dioxide, IPCC

## Abstract

**Background:**

Livestock play an important role in carbon cycling through consumption of biomass and emissions of methane. Recent research suggests that existing bottom-up inventories of livestock methane emissions in the US, such as those made using 2006 IPCC Tier 1 livestock emissions factors, are too low. This may be due to outdated information used to develop these emissions factors. In this study, we update information for cattle and swine by region, based on reported recent changes in animal body mass, feed quality and quantity, milk productivity, and management of animals and manure. We then use this updated information to calculate new livestock methane emissions factors for enteric fermentation in cattle, and for manure management in cattle and swine.

**Results:**

Using the new emissions factors, we estimate global livestock emissions of 119.1 ± 18.2 Tg methane in 2011; this quantity is 11% greater than that obtained using the IPCC 2006 emissions factors, encompassing an 8.4% increase in enteric fermentation methane, a 36.7% increase in manure management methane, and notable variability among regions and sources. For example, revised manure management methane emissions for 2011 in the US increased by 71.8%. For years through 2013, we present (a) annual livestock methane emissions, (b) complete annual livestock carbon budgets, including carbon dioxide emissions, and (c) spatial distributions of livestock methane and other carbon fluxes, downscaled to 0.05 × 0.05 degree resolution.

**Conclusions:**

Our revised bottom-up estimates of global livestock methane emissions are comparable to recently reported top-down global estimates for recent years, and account for a significant part of the increase in annual methane emissions since 2007. Our results suggest that livestock methane emissions, while not the dominant overall source of global methane emissions, may be a major contributor to the observed annual emissions increases over the 2000s to 2010s. Differences at regional and local scales may help distinguish livestock methane emissions from those of other sectors in future top-down studies. The revised estimates allow improved reconciliation of top-down and bottom-up estimates of methane emissions, will facilitate the development and evaluation of Earth system models, and provide consistent regional and global Tier 1 estimates for environmental assessments.

## Background

Livestock play an important role in agricultural carbon (C) cycling and are associated with large annual greenhouse gas emissions [1, 2]. The IPCC [3, 4] provides guidelines for bottom-up estimation of livestock emissions based on inventory, which have been employed at the global [5, 6] and national levels (e.g. annual reports to the United Nations Framework Convention on Climate Change). In inventory-based estimation of national livestock methane (CH_4_) emissions, annual standing populations of each animal type are multiplied by species- and region-specific emissions factors to obtain annual emissions quantities. The emissions factors are derived using sets of mathematical formulae with inputs that vary depending on regional livestock qualities and management (e.g. feed intake quantity and quality; milk production quantity; amount of energy used for growth, draft work, foraging, and pregnancy; and utilization of various manure management systems) [4].

The input information in the IPCC 2006 guidelines is based on literature reflecting earlier decades; e.g. sources listed for tables in Annexes 10.A1 and 10A.2 in [4] were published between 1976 and 2004, with most from the 1980s and 1990s. In at least some regions, this information no longer reflects the state of livestock. For example, in many industrialized or industrializing nations, management of manure in pits or lagoons, instead of on pasture or cropland, has become more prevalent [7, 8] and animals perform less draft work [9] than in earlier decades. For example, IPCC 2006 guidelines and recent publications based on them [10, 11] consider 12% of US dairy cattle manure to be managed in anaerobic lagoons, while more recent data from the US EPA [12] suggest that anaerobic lagoons are now much more widely used. Because CH_4_ emissions from anaerobic lagoons are calculated to be nearly twice the magnitude of those from aerobic systems per unit of manure input, these changes must be taken into account in new bottom-up inventories.

The IPCC 2006 default information is used to calculate bottom-up CH_4_ emissions in important global earth system simulation studies and environmental assessments [13, 14]. For example, in addition to reports from the IPCC [15], the US Environmental Protection Agency’s report on global emissions [16], IIASA’s greenhouse gas and air pollution interactions and synergies (GAINS) model [17], and the emissions database for global atmospheric research (EDGAR) [18] use IPCC 2006 default information, although the latter modifies cattle inputs based on carcass weight or milk productivity. IIASA’s RAINS model, an earlier source of global CH_4_ emissions used in a recent longer-term study along with EDGAR and EPA data [13], is based on IPCC 1996 [3] emissions factors [19]. Recent top-down estimates for the US, however, suggest that even revised methods based on IPCC guidelines underestimate livestock CH_4_ emissions in recent years at the national or state level [20–23]. Additionally, since 2007, global atmospheric concentrations of CH_4_ began increasing again after several stable years, and the ^13^C isotopic ratio of atmospheric CH_4_ concurrently become more negative; these changes may indicate increasing CH_4_ emissions from biogenic sources such as wetlands, rice paddies, and/or livestock in various global regions [24–28]. These changes and discrepancies illustrate the need for updated livestock CH_4_ emissions coefficients for bottom-up inventories.

Many factors are likely to impact recent livestock CH_4_ emissions quantities, such as the proportion of animals in large animal feeding operations that use various manure management systems; animal traits, such as body mass or productivity, which have changed with animal breeding and increased use of improved breeds; and animal feed quality and quantity, which may change over sub-annual and longer time periods. Here, we re-evaluated inputs used to calculate IPCC tier 1 CH_4_ emission factors for (1) enteric fermentation emissions in dairy cows and in meat/other cattle and (2) manure management emissions in dairy cows, meat/other cattle, and swine.

## Methods

### Revision of annual, per-animal CH_4_ emissions factors and other livestock C fluxes

The 2006 IPCC CH_4_ emissions factors were revised by (1) collecting updated regional input information (Tables 1, 2) and (2) following the Tier 2 equations for enteric fermentation and manure management CH_4_ emissions [4] with the updated inputs. This resulted in new emissions factors suitable for Tier 1 bottom-up inventory based estimates. To revise enteric fermentation emissions *factors for lactating dairy cows, for example*, Equations 10.2, 10.3, 10.4, 10.6, 10.8, 10.11, 10.13, 10.14, 10.16, 10.18b, and 10.21 were used with input from Tables 10.2, 10.4, 10.5, 10.8, 10.12, 10.A.1 [4] (Table 1). To revise manure CH_4_ emissions factors for dairy cows, meat/other cattle, and swine, Equations 10.23 and 10.24 were used with input from Tables 10.17, 10A-4, 10A-5, 10A-7, and 10A-8 [4] (Table 2). Some information on total dry matter intake and/or gross energy intake and manure production are also provided by IPCC; these quantities were also updated and used to create complete livestock C budgets (see below). Manure production for cattle was estimated from updated regional animal body weights, assuming that dairy cattle produce 2205 kg manure dry matter per animal unit per year, and meat/other cattle produce 1510 kg manure dry matter per animal unit per year [29]. Manure production for swine was estimated using IPCC 1996 regional swine body weight and manure production information [3] along with revised (recent) regional body weights, based on the approximation that intake scales with a three-fourths fractional exponent of body mass [30]:

**Table 1:** Dairy cow enteric fermentation emissions factor inputs

Region	Based on	Average animal body mass (kg, lactating cows only)		% Stall fed animals		Average milk production (kg/head/year)		Gross energy intake		Digestibility of feed (%)		Y_m_ (%)	
		IPCC 2006^a^	This study	IPCC 2006^a^	This study	IPCC 2006^a^	This study	IPCC 2006^a^	This study^a,b^	IPCC 2006^a^	This study	IPCC 2006^a^	This study
US and Canada	US	600	680 [12]	100	93 [12, 39]	8400	9732 [12, 39]	301.7	403.2	75	66.7 [12]	6.5	6 [70]
W. Europe	EU-15	600	624 [71]	100	77 [70]	6000	6935 [71]	275.4	304.9	70	70.2 [70]	6.5	6.53 [70]
E. Eur.–Ctrl. Asia	Russian Fed.	550	572 [4, 71]	100	80 [70]	2550	3898 [70]	232.5	233.9	60	68.6 [70]	6.5	6 [70]
Oceania	Australia	500	565 [70]	0	7 [70]	2190	5789 [70]	234.6	253	60	76.1 [70]	6.5	7.24 [70]
Latin America	Brazil	400	458 [72]	0	2 [73]	800	1208 [74]	179.3	207.1	60	60^c^	6.5	8 [74]
E.-S.E. Asia	China	350	500 [59, 75]	100	100 [58, 59, 76]	1650	3011 [36]	160.9	333.7	60	60^c^	6.5	7 [76]
Africa	Ethiopia	275	354.75^d^	100	50 [77]	475	687.2 [36, 77]	107.3	147.2	60	60^c^	6.5	8^e^
S. Asia	India	275	281 [78, 79]	100	70 [77]	900	1355.2 [36, 77]	135.4	160.2	55	55^c^	6.5	8^e^

Only lactating dairy cows are included in the dairy cow category. Reported values that included dairy calves, heifers, dry cows, and/or replacements were not used or were adjusted to reflect producing dairy cow populations

^a^Calculated using information in supplemental tables in Chapter 10 of IPCC 2006 [4]

^b^Net energy for growth and wool production are assumed to be zero for mature dairy cows

^c^Unchanged from information in supplemental tables in Chapter 10 of IPCC 2006 [4] due to lack of newer information

^d^Based on average increases found for Latin America and E.-S.E. Asia, due to lack of information

^e^As reported for Latin America, due to lack of information

**Table 2:** Cattle and swine manure management emissions input factors used for this study, as compared with those used/published by IPCC

Region^e^	Average animal body mass (kg)		Volatile solids (kg year^−1^)		B_o_ (m^3^ CH_4_/kg VS)		MCF for dry systems^f^		MCF liquid	
	IPCC 2006^d^	This study	IPCC 2006^d^	This study	IPCC 2006^d^	This study	IPCC 2006^d^	This study	IPCC 2006^d^	This study
Dairy cattle										
US and Canada	600	680^e^	1971	2703 [12, 39]	0.24	0.24 [12]	1.2	1 [12, 70]	20.0	29.5 [12]
W. Europe	600	624^e^	1862	1632 [70]	0.24	0.23 [70]	1.5	3.1 [70]	20.0	26 [70]
E. Eur.–Ctrl. Asia	550	572^e^	1643	1705 [70]	0.24	0.24 [70]	1.7	1.8 [70]	17.0	NA
Oceania	500	565^e^	1278	1033 [70]	0.24	0.24 [70]	1.4	1.4 [70]	50.0	35.0 [70]
Latin America	400	458^e^	1059	1507^h^	0.13	0.13^g^	0.9	0.9^g^	65.0	65.0^g^
E.–S.E. Asia	350	500^e^	1022	1602^h^	0.13	0.13^g^	1.7	2.0 [9]	19.0	20.0 [9]
Africa	275	355^e^	694	1071^h^	0.13	0.13^g^	1.9	1.9^g^	46.0	NA
S. Asia	275	281^e^	949	975^h^	0.13	0.13^g^	5.6	5.6^g^	55.0	55.0^g^
Meat/other cattle										
US and Canada	389	420 [70]	876	1059 [70]	0.19	0.20 [12, 39]^a^	1.0	1.4 [12, 39]	20.0	34.0 [12, 39]
W. Europe	420	401 [70]	949	726 [70]	0.18	0.19 [70]	1.5	4.5 [70]	20.0	29.0 [70]
E. Eur.–Ctrl. Asia	391	351 [70]	986	923 [70]	0.17	0.17 [70]	1.6	1.7 [70]	17.0	20.0 [70]
Oceania	330	376 [70]	1095	965 [70]	0.17	0.17 [70]	1.5	1.5 [70]	NA	NA
Latin America	305	323 [80, 81]	913	779^h^	0.10	0.10^g^	1.5	1.5^g^	NA	NA
E.–S.E. Asia	319	259 [59]	840	546^h^	0.10	0.10^g^	1.2	1.2^g^	NA	NA
Africa	173	173^g^	548	548^g^	0.10	0.10^g^	1.7	1.7^g^	NA	NA
S. Asia	110	208 [78]	511	676^h^	0.10	0.10^g^	5.9	5.9^g^	55.0	55.0^g^
Swine										
US and Canada	61	60 [70]	99	106 [70]	0.48	0.48 [70]	1.5	1.5 [12, 70]	20.0	30.7 [12, 70]
W. Europe	50	76 [70]	110	113 [70]	0.45	0.41 [70]	1.8	4.4 [70]	20.0	26.5 [70]
E. Eur.–Ctrl. Asia	50	55 [70]	110	168 [70]	0.45	0.45 [70]	2.3	2.0 [70]	17.0	20.0 [70]
Oceania	45	61 [70]	102	113 [70]	0.45	0.45 [70]	1.4	1.0 [70]	65.0	65.0 [70]
Latin America	28	60 [82]	110	106 [82]	0.29	0.48 [82]	1.5	1.5 [82]	60.0	60.0 [82]
E.–S.E. Asia	28	60 [62, 83]	110	110^g^	0.29	0.29^g^	1.0	1.0^g^	19.0	19.0^g^
Africa	28	28^g^	110	110^g^	0.29	0.29^g^	1.7	1.7^g^	60.0	60.0^g^
S. Asia	28	28^g^	110	110^g^	0.29	0.29^g^	3.0	3.0^g^	55.0	55.0^g^

Region	MCF lagoon		MCF digester/other	% of manure managed in dry systems a		% of manure managed in liquid and deep pit systems		% of manure managed in lagoon systems		% of manure managed in digesters/other	
	IPCC 2006^d^	This study	This study	IPCC 2006^d^	This study	IPCC 2006^d^	This study	IPCC 2006^d^	This study	IPCC 2006^d^	This study
Dairy cattle											
US and Canada	70.0	71 [12, 39]	NA	58	42 [12, 39]	30	24.3 [12, 39]	15	33.6 [12, 39]	0	0 [12]
W. Europe	70.0	42 [70]	18.0 [70]	64	54 [70]	36	41 [70]	0	0 [70]	0	5 [70]
E. Eur.–Ctrl. Asia	66.0	NA	NA	83	100 [70]	18	0 [70]	0	0 [70]	0	0 [70]
Oceania	78.0	90.0 [70]	NA	84	95 [70]	1	0 [70]	16	5 [70]	0	0 [70]
Latin America	78.0	NA	NA	99	99 [73]	1	1 [73]	0	0 [73]	0	0 [73]
E.–S.E. Asia	68.0	NA	10.0^d^	56	66 [59]	38	25 [9]	4	0 [59]	2	9 [59]
Africa	79.0	NA	NA	100	100^g^	0	0^g^	0	0^g^	0	0^g^
S. Asia	79.0	NA	10.0^d^	99	80 [84]	1	0 [84]	0	0 [84]	1	20 [84]^b^
Meat/other cattle											
US and Canada	NA	NA	NA	100	100 [12, 39]	0	1 [12, 39]^c^	0	0 [12, 39]	0	0 [12, 39]
W. Europe	NA	NA	NA	75	72 [70]	25	26 [70]	0	0 [70]	0	3 [70]
E. Eur.–Ctrl. Asia	NA	NA	NA	78	86 [70]	23	14 [70]	0	0 [70]	0	0 [70]
Oceania	NA	NA	NA	100	100 [70]	0	0 [70]	0	0 [70]	0	0 [70]
Latin America	NA	NA	NA	100	100^g^	0	0^g^	0	0^g^	0	0^g^
E.–S.E. Asia	NA	NA	NA	100	98 [59]	0	0 [59]	0	0 [59]	0	2 [59]
Africa	NA	NA	NA	100	100^g^	0	0^g^	0	0^g^	0	0^g^
S. Asia	NA	NA	NA	99	99^g^	1	1^g^	0	0^g^	0	0^g^
Swine											
US and Canada	70.0	72.2 [12, 39]	NA	8	6 [12, 39]	59	58 [12, 39]	33	36 [12, 39]	0	0 [12]
W. Europe	70.0	41.7 [70]	16.5	22	13 [70]	70	58 [70]	9	3 [70]	0	26 [70]
E. Eur.–Ctrl. Asia	66.0	NA	NA	72	37 [70]	25	63 [70]	3	0 [70]	0	0 [70]
Oceania	78.0	90.0 [70]	10.0	46	23 [70]	0	0 [70]	54	76 [70]	0	1 [70]
Latin America	79.0	79.0 [82]	NA	93	30 [82]	8	0 [82]	0	70 [82]	0	0 [82]
E.–S.E. Asia	68.0	68.0^g^	10.0	54	28 [63, 85–87]	40	55 [63, 85–87]	0	0^g^	7	18 [63, 85–87]
Africa	79.0	NA	NA	94	94^g^	6	6^g^	0	0^g^	0	0^g^
S. Asia	79.0	NA	NA	69	69^g^	22	22^g^	9	9^g^	0	0^g^

*NA* not applicable

^a^B_o_ for US meat/other cattle was calculated as the weighted average of on-feed and not-on-feed live-animal biomass. We estimated that 17.2% of US meat/other cattle live animal mass is on-feed in 2012, based on reported body masses and populations [39]. Values of B_o_ for these populations was taken from [12]

^b^CH_4_ emissions from the estimated 20% of manure that is burned for fuel in this region were considered similar to emissions from manure treated in anaerobic digestors for lack of specific information; CH_4_ and/or volatile solids assumed to be oxidized to CO_2_ when burned

^c^Table A-206 in [12] states “because manure from beef feedlots…may be managed for long periods of time in multiple systems (i.e. both drylot and runoff collection pond), the percent of manure that generates emissions is greater than 100%.”

^d^Calculated using information in supplemental tables in Chapter 10 of IPCC 2006 [4]

^e^See Table 1 for the country/countries on which regional values are based, and for dairy cattle weight sources

^f^Dry systems include dry lot, pasture/range, solid, daily spread, burned, and pit storage <1 month

^g^Unchanged from information in supplemental tables in Chapter 10 of IPCC 2006 [4] due to lack of newer information

^h^Calculated using the updated information presented here in Eq. 10.24 in Chapter 10 of IPCC 2006 [4]

1 $${\text{manure}}\text{-}{\text{production}}_{\text{revised}} = {\text{ manure}}\text{-}{\text{production}}_{{ 1 9 9 6 {\text{IPCC}}}} \times \, \left[ {{\text{weight}}_{\text{revised}} /{\text{weight}}_{{ 1 9 9 6 {\text{IPCC}}}} } \right]^{0. 7 5}$$

To evaluate our bottom-up approach to estimating C stocks and fluxes, the equations and default inputs were first used to recalculate the IPCC 2006 CH_4_ emissions factors. Literature search results were then used to revise inputs and recalculate these equations.

For dairy cow enteric fermentation CH_4_ emissions factors, revisions focused on changes in mature animal weight, percent of animals that are stall fed as opposed to grazing/ranging for feed, annual milk productivity, changes in total feed intake, and on reported values of Y_m_ (the CH_4_ conversion factors for feed energy intake during enteric fermentation). For these calculations, we assumed that mature lactating dairy cows do not gain or lose weight, so that net energy for growth takes a value of zero. For enteric fermentation CH_4_ emissions from meat/other cattle, we use recently reported emissions factors from national UNFSCCC reports where available, and where such information was not available, we calculated revised factors based on changes in animal body weight only. This approach was taken due to the complexity and variability in important management factors for meat cattle, particularly in industrialized systems (e.g. type of diet provided, timing of placement from pasture to feedlot, slaughter age and weight).

For manure management CH_4_ emissions factors, revisions focused on changes in animal weight at slaughter, changes in total feed intake and feed digestibility, and changes in the percentage of manure managed in various manure management systems (e.g. deposited on pasture, drylot storage, short-term pit storage, long-term anaerobic lagoon treatment), and MCFs (methane conversion factors, the CH_4_ conversion factors for manure volatile solids during manure storage and/or treatment) for different manure management systems at various temperatures. Because of the difficulty in obtaining recent information for all regions of the world, we did not revise B_o_ (the amount of CH_4_ produced per quantity of manure volatile solids). Manure management CH_4_ emissions factors were revised for (1) lactating dairy cattle; (2) meat/other cattle (encompassing meat and dairy calves and heifers and all other cohorts of non-lactating cattle grown for slaughter, replacement, breeding, or other purposes, weighted using mean weights and reported population cohorts), and (3) swine (encompassing farrowing sows, nursing piglets, and feeders, weighted using mean weights and reported population cohorts). For meat/other cattle in the US, where in recent years animals weighed 27–45 kg at birth [31], were weaned at ~260 kg [31], were placed on feedlots at ~317 kg [32], and were slaughtered at ~610 kg [33], the amounts of manure managed on pasture and on feedlot were weighted by average cohort masses accordingly.

### Uncertainty analysis

We employed IPCC 2006 Uncertainty Approach I: Propagation of Error [34] to arithmetically combine the uncertainties associated with livestock carbon fluxes of interest:

Where uncertain quantities are to be combined by multiplication, the standard deviation of the sum will be the square root of the sum of the squares of the standard deviations of the quantities that are added, with the standard deviations all expressed as coefficients of variation, which are the ratios of the standard deviations to the appropriate mean values…Where uncertain quantities are to be combined by addition or subtraction, the standard deviation of the sum will be the square root of the sum of the squares of the standard deviations of the quantities that are added with the standard deviations all expressed in absolute terms … [34]

When the uncertainties being combined can be considered independent, their standard deviations or coefficients of variation are added in quadrature (i.e. the square root of the sum of the squares of each standard deviation or coefficient of variation) [35]. This has the effect of reducing overall propagated uncertainty. We added in quadrature when propagating uncertainties within a livestock type, because we independently assembled separate estimates of the various carbon fluxes and their uncertainties (e.g. intake, manure production, milk production, CH_4_ emissions) except for CO_2_, which is calculated by subtraction. We then used these uncertainties to calculate fractional standard deviations (equal to the coefficient of variation, the standard deviation divided by the mean value) for each per-animal carbon flux quantity in each global region. However, when combining uncertainties across livestock types within a nation or from multiple nations to the regional or global level, the uncertainties were simply added (not in quadrature), because these estimates are not independent [35]—i.e. the livestock in all nations within a region share the same carbon flux estimates, emissions coefficients, and uncertainties, and all livestock within a nation share many regional attributes. Using the arithmetic sum, as opposed to adding in quadrature, results in larger uncertainties, which may be considered more conservative.

Uncertainty on all non-CH_4_ quantities is derived from the coefficients of variation (the standard deviation/mean value of the quantity) that we calculated for these quantities in previous work [2]. Uncertainty on IPCC livestock CH_4_ emissions factors is given as ±30% [4], and is defined as representing ±1.96 times the standard deviation of the mean [34]. In order to be combined mathematically [34, 35] with our estimates of uncertainty on other C fluxes, we used 15.3% (30% divided by 1.96) as the uncertainty for all calculated CH_4_ quantities.

### Derivation of annual livestock C fluxes, including emissions of CO_2_ and CH_4_

We assumed a linear transition from IPCC 2006 emissions to revised emissions factors during the years 1990–2012:

2 $${\text{f}}_{\text{yeari}} = {\text{f}}_{\text{IPCC}} + \, \left( {{\text{ f}}_{\text{revised}} - {\text{f}}_{\text{IPCC}} } \right) \cdot \left( {{\text{Y}}/ 2 2 { }} \right)$$

where f_yeari_ is the flux of CH_4_, feed, or other C containing quantity per animal in the year of interest; f_IPCC_ is the flux of CH_4_, feed, or other C quantity per animal given or calculated from data provided by 2006 IPCC guidelines [4]; f_revised_ is the revised flux of CH_4_, feed, or other C quantity per animal (resulting from this work); and Y is equal to 0 for years before 1990, to (year—1990) for 1990–2012; and to 22 for years after 2012.

Livestock carbon dioxide (CO_2_) emissions associated with respiration were estimated as the deficit between the C contained in annual livestock feed intake and the sum enteric fermentation CH_4_ emissions, production of milk or eggs, and manure production. Similarly, CO_2_ emissions associated with manure management were estimated as the difference between total manure C production and manure management CH_4_ emissions, assuming that all manure C is emitted as either CH_4_ or CO_2_ within one year of production.

### Livestock populations

Annual national livestock populations of meat and milk-producing cattle, meat and milk-producing buffaloes, meat and egg-laying chickens, swine, sheep, turkeys, ducks, geese and guinea fowl, goats, horses, mules, asses, camels, and other camelids (i.e. llamas and alpacas) were compiled for years 1961–2013 from FAOSTAT [36]. Annual producing populations of egg-laying chickens and milk-producing cattle and buffalo were subtracted from conspecific total populations to estimate populations raised for meat production. For all calculations made here, the dairy cattle livestock populations include only milk-producing mature dairy cows; calves, heifers, breeding steers, and any other dairy cattle ‘replacements’ are categorized with meat/other cattle. For nine large countries (Argentina, Brazil, Canada, Chile, China, India, Kazakhstan, Mexico, and the Russian Federation), state- or province-level livestock population data were compiled for available years between 2000 and 2011 [37, 38], and used to improve the spatial distribution of inventory data. For the United States, livestock populations were refined to the county level using National Agricultural Statistical Service Census and Survey data [39]. Livestock in all other nations of the world are constrained at the national level only.

### Livestock C fluxes and CH_4_ emissions

Accounting of livestock C fluxes was conducted as described in Wolf et al. [2]. Annual per-animal dry weight feed intake, dry weight manure production, manure C content, milk and egg production C, and manure management and enteric fermentation CH_4_ emissions are from IPCC [4] or were estimated from existing literature. Livestock dry matter intakes were assumed to be 44% C by weight. The difference between total livestock feed intake C and total C produced or emitted by live animals (i.e. the sum of C contained in manure, enteric fermentation CH_4_, and milk and eggs) approximates the amount of C respired in the form of CO_2_ over a given year, excluding C stored in livestock biomass. Although herd sizes do change over time, C stored in livestock biomass is assumed constant in this effort. Similarly, the difference between total manure C content and manure management CH_4_ provides an estimate of CO_2_ released by livestock manure management, all of which is assumed to be emitted in the same year of manure production.

### Estimating livestock consumption of fodder and forage

For purposes of tracking the use of all harvested crop C and estimating amounts of livestock forage, total livestock feed was disaggregated into fodder (i.e. biomass harvested by humans from croplands) and forage (i.e. biomass grazed or scavenged by livestock from non-cropland sources) [2]. Fodder was further subdivided into (a) market feed items derived from primary harvests (e.g., grains, brans, crop by-product feeds), derived from FAO [36] (food balance: commodity balances, crops primary equivalent, feed category), (b) hay and fodder crops (e.g., harvested quantities of alfalfa, clovers, grasses, corn and sorghum silage) derived from FAO [36] (production: crops, crops primary list), including maize, alfalfa, and other grains, grasses, legumes, roots, and vegetables denoted as produced for forage and/or silage; category no longer available), and (c) crop residue feed, consisting of crop residue collected from the field for livestock feed, estimated from annual production of several utilized crops [2]. Annual national quantities of all market feed items and hay crops available were converted into units of C using fractional item-specific dry weights and C contents [2]. The crop residue feed quantities were estimated by applying crop-specific regional percentages of residues collected for feed [40] to the crop- and country-specific estimates of annual residue production. Total annual available fodder per nation is the sum of market feeds, hay and fodder crop production, and crop residues collected for feed. At the national level, annual available fodder was subtracted from total livestock feed intake requirement (calculated from national annual populations and per-animal feed intake values) to approximate national livestock forage intake, including grazing and scavenging. Because national quantities of market feeds and hay crops were not available for years after 2011 at the time of download, fodder and forage intake for 2012 and 2013 were estimated using average available quantities for each country over 2005–2011.

### Downscaling and spatial distribution of C fluxes

Livestock C fluxes were downscaled and spatially distributed to 0.05 × 0.05 degree resolution using the MODIS Land Cover Type 5 data product for year 2005, following methods documented by West et al. [41] and Wolf et al. [2]. Downscaling started with the reconciling of land class areas between satellite-based land cover in 2005 and crop harvest area inventory data in each year from 2000 to 2011. Cropland area in 2005, based on MODIS, was compared to the sum of area inventoried for harvest per geopolitical region. The MODIS cropland areas were then adjusted to equal the sum of harvested areas for respective geopolitical regions and years. Cropland area was expanded or contracted as necessary, using a global kernel density representing the combined density of cropland and distance of each grid-cell to the nearest cropland region. Based on reconciled land cover information within each nation, state or province, or county, a separate amount of area was allocated to livestock. The livestock area requirement per nation, state/province, or county was derived from the livestock population therein, along with estimated area per animal required for each livestock type, for housed and free-ranging animals, and regional estimates of the proportion of animals that are free-ranging. Livestock were spatially distributed to grasslands, based on the livestock area requirement, per nation, state/province, or county. If there was insufficient grassland area, livestock were then distributed to shrubland areas. If grassland and shrubland areas together were smaller than the estimated required livestock area, the livestock area requirement was reduced to a smaller housed-animal area requirement value, thereby increasing livestock density. Respective carbon fluxes were subsequently applied to spatial livestock distributions.

## Results

### Revised livestock emissions factors

The revised emissions factors calculated here are greater than those given by IPCC 2006 for many, but not all, livestock types and regions (Table 3). The information we assembled to revise emissions factors highlights important recent changes in regional livestock systems. Mature dairy cattle body mass and milk productivity were greater in all global regions than IPCC 2006 default values, although the magnitude of increase varied (Table 1). Revised enteric fermentation emissions factors for dairy cows range from 7% smaller (E. Europe and W. and Central Asia) to 125% larger (E. and S.E. Asia) than IPCC 2006 emissions factors (Table 3). Dairy manure management strategies changed along with increasing dairy cow body mass and productivity (Table 2). This resulted in more variable changes in manure management emissions factors among global regions than enteric fermentation emissions factors for dairy cows. Changes in dairy cow manure management emissions factors ranged from a 68% decrease in Oceania to a 158% increase in the US and Canada region (Table 3).

**Table 3:** Emissions factors (kg CH_4_ -animal^−1^ year^−1^) as given by IPCC 2006 [4] and as revised resulting from this study

Region	Dairy cow enteric fermentation		Meat and other cattle enteric fermentation		Dairy cow manure management		Meat and other cattle manure management		Swine manure management	
	IPCC 2006^a^	This study^b^	IPCC 2006^a^	This study^d^	IPCC 2006^a^	This study^b^	IPCC 2006^a^	This study^b^	IPCC 2006^a^	This study^b^
US–Canada	128	158.7	53	58.8	53	137.0	1	2.4	12	15
W. Europe	117	130.6	57	46.5	25	31.0	7	9.9	7	6.7
E. Europe	99	92.1	58	56.1	12	4.9	6	4.5	3	6.8
Oceania	90	120.1	60	71.9	29	9.4	2	1.6	13	23.6
Latin America	72	108.6	56	57.9	1	2.0	1	0.8	1	19
E.–S.E. Asia	68	153.2	47	42.4	10	10.1	1	0.4	2	2.7
Africa	46	77.2	31	31.0	1	1.8	1	1^c^	1	1^c^
S. Asia	58	62.4	27	41.6	5	5.5	2	3	5	5^c^

^a^IPCC 2006 [4], Chapter 10: Agriculture, supplemental tables

^b^Calculated using the updated information presented here in the equations in IPCC 2006 [4], Chapter 10

^c^Emissions factors not modified from IPCC 2006 [4] due to sparse information

^d^2014 National Inventory Submissions to the UNFCCC, CRF, Table 4. A reported for all non-dairy cattle in year 2012 (US, EU15, Russian Fed., and Australia); or calculated using IPCC 2006 [4] defaults except for revised body weights listed in Table 2

In contrast to the increases in mature dairy cow body mass, we found that body mass at time of slaughter for meat/other cattle decreased in several regions (Table 2). The mature weights of producing dairy cows are determined by breed/genetics and nutritional status of the animals. While this is also true for meat/other cattle, slaughter weights for meat animals are also determined by management decisions, and as such may vary with changing economic or environmental factors (e.g. weather extremes, feed costs, or meat prices and demand). For meat cattle in many regions, external factors also influence the weight at which grazing animals are placed on feedlots to be grain-finished—with very large differences in manure management CH_4_ emissions between these situations (Table 2). Changes in emissions factors for enteric fermentation in meat/other cattle ranged from an 18% decrease (W. Europe) to a 54% increase (E. and S.E. Asia). Manure management CH_4_ emissions factors for meat/other cattle are overall much smaller than those for dairy cows, and the IPCC 2006 default factors are rounded to the nearest integer value (e.g. “1”). Therefore, some of the changes reported here result merely from inclusion of additional significant digits. Given the large global populations of meat/other cattle, these small changes are nevertheless important. Revision of manure management emissions factors for meat/other cattle resulted in variable changes among regions, ranging from a 60% decrease (E. and S.E. Asia) to a 140% increase (US and Canada).

Changes in swine manure management emissions factors, relative to IPCC 2006 reported values, range from −4% (W. Europe) to +1800% in Latin America. The latter large increase is due to modernization of swine production in that region, including use of improved breeds with larger potential body mass, changing animal diet, and in particular a shift from dry manure management systems to anaerobic lagoons.

### Revised global livestock C fluxes

**Fig. 1:**
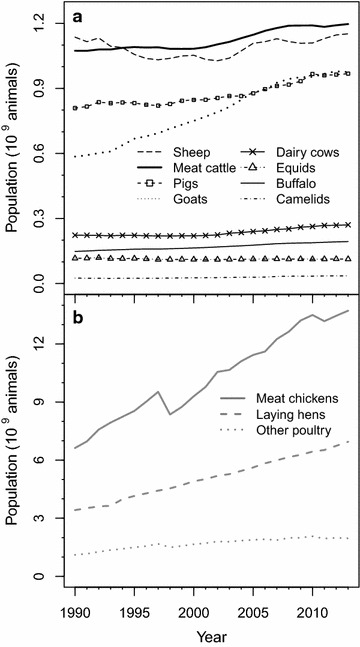
Annual global populations of mammalian livestock (**a**) and poultry (**b**), in billions, for 1990–2013 period

**Fig. 2:**
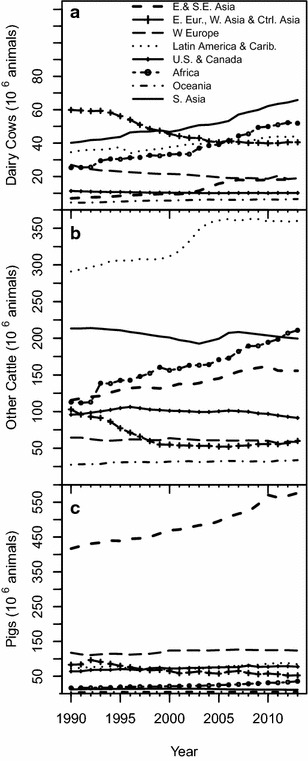
Annual regional populations of dairy cattle, meat/other cattle, and swine

Fluctuations in annual livestock populations [FAO, 36] play a large role in the magnitude of C fluxes associated with livestock, including CH_4_ emissions. Global populations of most livestock species did not change greatly over the years between 1990 and 2013, except for goats and chickens (Fig. 1). However, when separated by region, changes in the distribution of global cattle and swine populations are apparent (Fig. 2). For dairy cows, meat/other cattle, and swine, populations in W. Europe and US and Canada regions remained steady or declined slightly over the years in this study. In contrast, meat/other cattle populations increased dramatically in Latin America during the early 2000s, and the already large swine population in E. and S.E. Asia has continued to increase in recent decades.

**Fig. 3:**
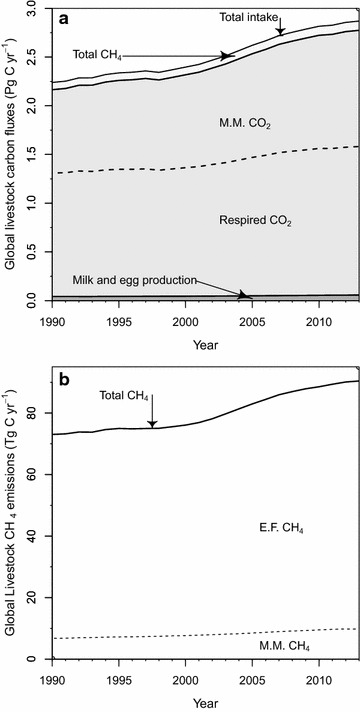
Revised annual global livestock carbon fluxes, 1990–2013: carbon contained in all fluxes associated with livestock (**a**), and carbon contained in methane emissions associated with manure management (M.M.) and enteric fermentation (E.F.). Note different units

**Fig. 4:**
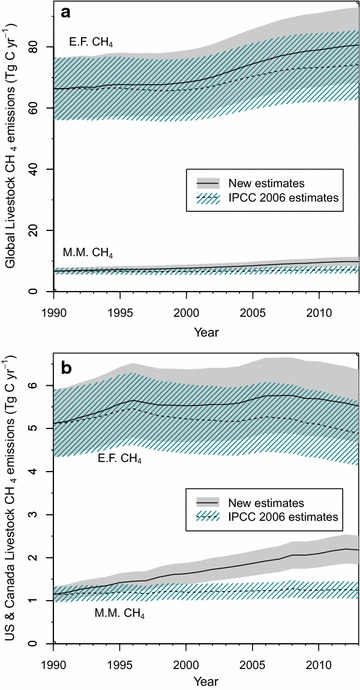
Comparison of enteric fermentation (E.F.) and manure management (M.M.) methane emissions estimated using IPCC 2006 and revised emissions factors resulting from this study, for the globe (**a**), and the US and Canada region (**b**)

Total livestock CH_4_ emissions account for ca. 3% of total livestock C fluxes (Fig. 3; Table 4). Nevertheless, estimating livestock CH_4_ emissions with our revised emissions factors results in discernably larger emissions relative to calculations made using IPCC 2006 emissions factors. Revised global total CH_4_ C emission quantities for 2011 are 89.4 ± 13.7 Tg C (119.1 ± 18.2 Tg CH_4_), an increase of 11% over estimates made using IPCC 2006 emissions factors. This change encompasses an 8.4% increase in enteric fermentation CH_4_ C and a 36.7% increase in manure management CH_4_ C (Fig. 4a). In certain regions, these changes are more pronounced, such as in the US and Canada (Fig. 4b), where 2011 total livestock CH_4_ emissions were 24.2% greater than when calculated with IPCC 2006 emissions factors, including a 12.3% increase in enteric fermentation CH_4_ C and a 71.8% increase in manure management CH_4_ C (Fig. 4b).

**Table 4:** Livestock C fluxes by region for year 2011

2011 livestock C fluxes ± 1 SE	Region								
	Africa	E.–S.E. Asia	E. Europe, W. Asia, and Central Asia	Latin America	Oceania	S. Asia	US and Canada	W. Europe	Globe
Intake C (Tg C)	415 ± 52.2	610.7 ± 67.8	261 ± 31.8	559.5 ± 80.4	76.6 ± 10.5	451.2 ± 66.7	256.1 ± 34	195.7 ± 26.3	2825.7 ± 369.8
Manure production C (Tg C)	176.4 ± 30.8	219.8 ± 35.4	111.4 ± 19.2	259.1 ± 49.5	34.7 ± 6.2	197.4 ± 37.8	99.8 ± 18.4	82.5 ± 15	1181.1 ± 212.4
Enteric Fermentation CH_4_ C (Tg C)	12.69 ± 1.94	10.5 ± 1.61	6.16 ± 0.94	20.26 ± 3.1	2.97 ± 0.45	16.89 ± 2.58	5.64 ± 0.86	4.56 ± 0.7	79.67 ± 12.19
Manure Management CH_4_ C (Tg C)	0.45 ± 0.07	1.53 ± 0.23	0.73 ± 0.11	1.63 ± 0.25	0.21 ± 0.03	1.39 ± 0.21	2.16 ± 0.33	1.58 ± 0.24	9.68 ± 1.48
Total CH_4_ C	13.14 ± 2.01	12.03 ± 1.84	6.89 ± 1.05	21.89 ± 3.35	3.18 ± 0.49	18.29 ± 2.8	7.79 ± 1.19	6.14 ± 0.94	89.35 ± 13.67
Milk and egg production C (Tg C)	3.41 ± 0.84	8.34 ± 1.95	9.52 ± 2.35	4.75 ± 1.15	2.32 ± 0.58	9.9 ± 2.46	7.27 ± 1.79	10.21 ± 2.53	55.73 ± 13.65
Respiration CO_2_ C (Tg C)	222.5 ± 87.7	372 ± 108.3	133.9 ± 55.4	275.4 ± 137.1	36.6 ± 18.4	227 ± 112	143.3 ± 56.3	98.5 ± 45.4	1509.2 ± 620.6
Manure management CO_2_ C (Tg C)	176 ± 31	218.3 ± 35.8	110.7 ± 19.5	257.4 ± 50	34.5 ± 6.3	196 ± 38.2	97.7 ± 19	80.9 ± 15.5	1171.4 ± 215.3
Available fodder (Tg C)	83	260.5	212.7	157.3	8.6	231.6	136.6	156.9	1247.2
Unused/waste fodder^a^	4.9	8.6	38.4	0	0	0.5	0	19.4	71.8
Fodder intake^a^ (Tg C)	78.1	251.9	174.2	157.3	8.6	231.1	136.6	137.6	1175.4
Forage intake^a^ (Tg C)	336.9	358.7	86.8	402.1	68	220.1	119.5	58.2	1650.3
% of intake from forage	81.2	58.7	33.2	71.9	88.8	48.8	46.7	29.7	58.4

^a^Unused/waste fodder occurs when the amount of available fodder C is greater than livestock feed requirements per nation in a given year. In 2011, this occurred in: the Czech Republic, Denmark, Egypt, Germany, Hungary, Japan, Kazakhstan, Malaysia, the Russian Federation, Slovakia, Sri Lanka, and the Ukraine, and could be due to waste, stockpiling, misreporting, or other errors. Actual fodder losses per nation were not estimated

**Fig. 5:**
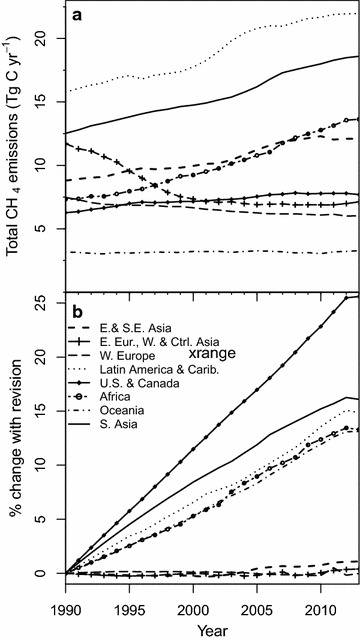
Revised total livestock methane emissions by region (**a**) and percent change in annual emissions relative to calculations made based on IPCC 2006 emissions factors

Over the 1990–2013 period, total livestock CH_4_ C emissions exhibit contrasting dynamics among global regions (Fig. 5a) due to trends in livestock populations (Fig. 2) as well as to revision of emission factors (Table 3). The changes in total livestock emissions relative to IPCC 2006 calculations vary by region (Fig. 5b). The largest changes are seen in the US and Canada region, despite declines in dairy (−7.7%) and meat/other cattle (−5.8%) populations there (Figs. 2, 5b).

**Fig. 6:**
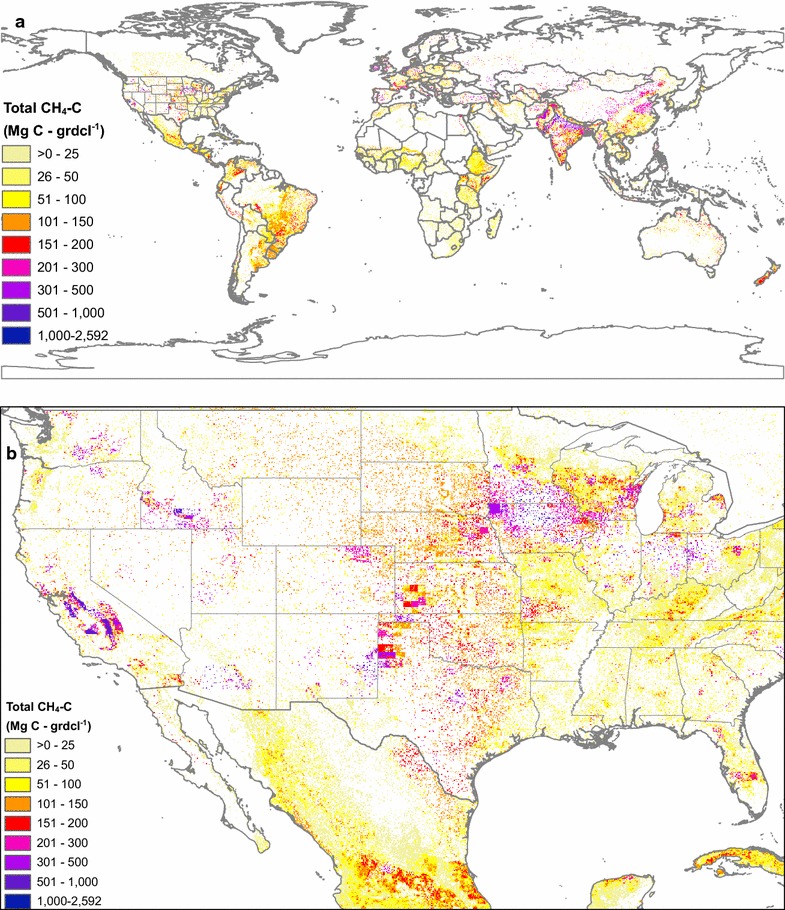
Total livestock methane emissions in 2011, downscaled to 0.05 × 0.05° resolution, for the globe (**a**) and detail for the western US (**b**)

**Fig. 7:**
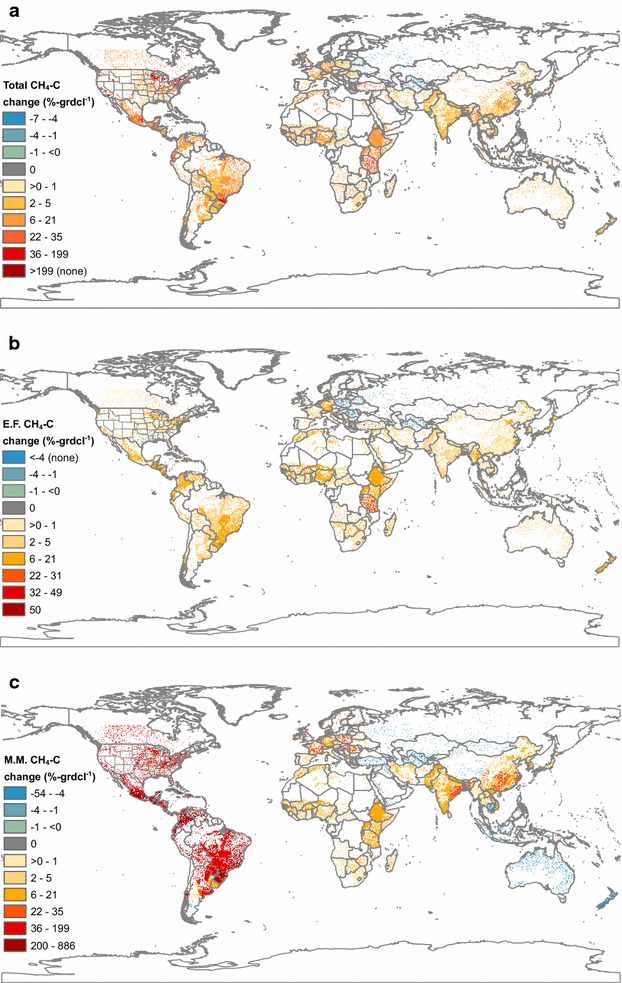
Percent change in global livestock methane emissions with revision, downscaled to 0.05 × 0.05° resolution, for the total (**a**), enteric fermentation (E.F.) (**b**), and manure management methane (M.M.) emissions (**c**), in 2011

Livestock C fluxes, including solids (i.e. feed intake and manure production) and gases (i.e. respiration and manure management CO_2_ C, and enteric fermentation and manure management CH_4_ C) are downscaled and mapped at 0.05 × 0.05° resolution, in both g C per m^2^ and Mg C per 0.05° gridcell formats for years 2000–2013. The maps show the interplay between regional livestock characteristics and emissions factors, national, state, or county level cohorts of various livestock species and types, and local densities of livestock. For livestock CH_4_ C fluxes in 2011 (Fig. 6), the percent change from calculations made using IPCC 2006 emissions factors are also downscaled and mapped (Fig. 7).

### Revised livestock forage intake and global livestock C budget

**Fig. 8:**
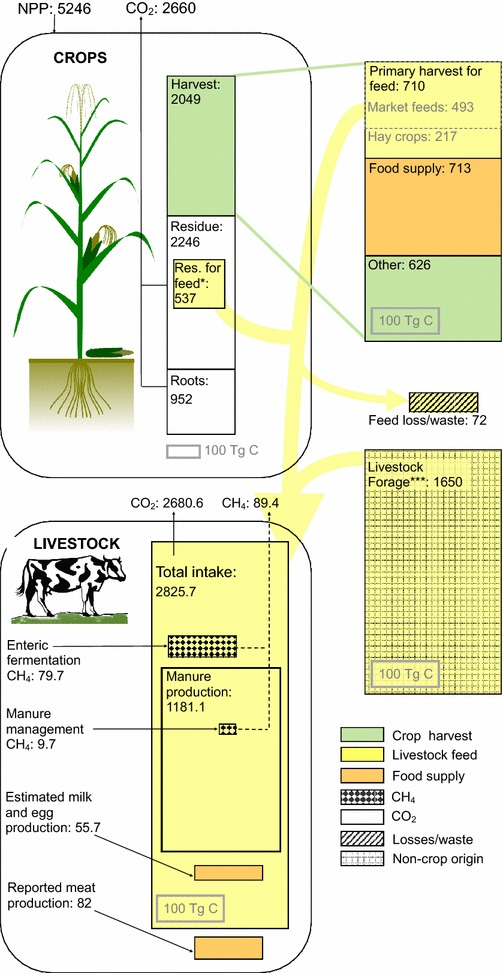
Revised livestock C budget for 2011. All non-harvested crop biomass C, and all manure C not emitted as CH_4_, are assumed to be decomposed and respired as CO_2_ by decomposing organisms within the same year as production

We show the revised global livestock C budget for 2011 in Fig. 8, using boxes with areas proportional to the magnitudes of the C flux represented. Our revised data are available through 2013, but because livestock fodder items were not available beyond the year 2011 at the time of data download [36], we estimated 2012 and 2013 fodder quantities based on 2005–2011 average availability. Crop NPP, primary (main crop) harvest C, and residue collected for feed were calculated as in Wolf et al. [2]; reported meat production is converted to Tg C from FAO reports [36] of total global meat production in 2011 (292 Tg of meat entering food supply) multiplied by conversion factors to estimate C content [2]. Estimated milk and egg production are the result of our calculations, based on estimated per-animal production by region. Our global value of 55.7 Tg C is similar to the value of 55.2 Tg C obtained by multiplying FAO reported global production [36] (743 Tg of milk, 71 Tg of eggs), and conversion factors for milk and egg C content in 2011 [2]. In comparison to primary crop harvest, crop residue harvest, and the quantity of livestock-based food produced, the magnitude of livestock fodder and forage consumption is apparent. Emissions of CO_2_ associated with livestock respiration and manure management are also shown, which are calculated by subtraction of all other fluxes from total intake or total manure production at the per-animal level, assuming static standing live populations with no net change in biomass across years.

Note that the ‘market feed’ category includes primary crop products as well as crop by-products that are unsuited or undesirable for human consumption, such as distillers grains (a by-product of bioethanol production) and various oil-crop extraction by-products (e.g. oil seed meal or cake). The C contained in and used for production of biofuels is included in the harvest/other uses box, but C contained in biofuel by-products sold and consumed as feed are pushed back into the primary harvest for feed/market feeds box; this results in a smaller total amount of C devoted to biofuels than in calculations that do not account for use of by-products in livestock feeds [2].

**Fig. 9:**
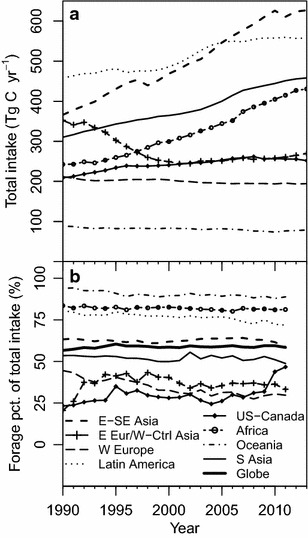
Revised total livestock feed intake carbon (**a**) and percent supplied by forage (**b**) by region

For most livestock types and regions, default livestock body weights and total feed requirements increased in our revision (Tables 1, 2). Reported amounts of annual available fodder, however, did not change [36]. In our accounting, the gap between total feed requirements and available fodder in each nation, if any, is filled by forage intake (i.e. grazing). Therefore, our revision of total livestock feed requirements also necessitated revision of livestock forage intake and the percentage of total livestock C intake supplied by forage. The revised percent of global livestock intake supplied by forage was 58.4% in 2011, reflecting 1.65 Pg C of forage intake from global rangelands (Table 4; Fig. 9b). These percentages are similar to estimates reported by other researchers; Bouwman et al. [42] estimated 59.2% of total livestock intake from forage in 1990 (our value is 56.6% for that year), and Krausman et al. [40] estimated 54.5% in 2000 (our value is 58.6%). When feed intake requirements were calculated using IPCC 2006 or IPCC 1996 [2, 3] livestock total intake values, the estimates for 2011 were 55.2 and 52.4%, respectively.

**Fig. 10:**
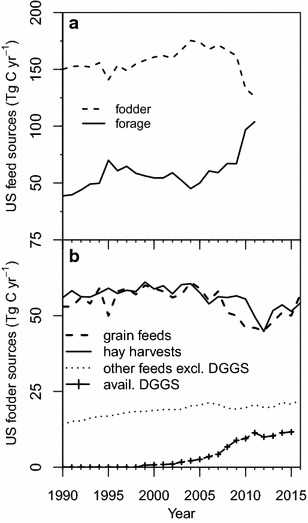
Detail of US livestock intake of fodder and forage (**a**) and US fodder sources (**b**)

Although the percentage of forage intake increased based on our revision, the global average percentage did not change greatly over the 1990–2011 time period, ranging between 56.6 and 60.7% (Fig. 9b). This suggests that, at the global level, amounts of forage and fodder intake C have increased apace over this time period to meet increasing total livestock intake requirements. This is also true in most regions during this time period, except for E. Europe and West and Central Asia after the breakup of the Soviet Union. However, in the US and Canada, the percentage of intake from forage increased sharply in 1995 and again after 2009, with the 2011 value (45.1%) doubled from 2005 (22.4%). Additional data from USDA (annual grain quantities fed, hay harvests, and by-product feed quantities excluding distiller’s grains) [43] and from the Renewable Fuels Association (annual quantities of distiller’s grains by-products from bioethanol production in the US, decreased by the estimated one-third that is exported annually) [44] were converted to units of C [2] to provide approximate annual amounts of available fodder in the US (Fig. 10). These data support the observed increases in percent livestock intake from forage in those years. The spike in and after 1995 can be attributed to drought in the Midwest US and other factors [45]. Uncertain harvests in the US and in E. Europe, along with the increasing use of corn for bioethanol production, may be the causes of the sharp increase after 2009 [46]. Corn prices, which averaged $2.75 per bushel in the 2000s, jumped to an average of $6.10 in 2010–2013 [43]. The jump in corn prices could have driven farmers to delay moving cattle from pasture to feedlots, without deterring the subsidized and mandated production of bioethanol in the US. In addition to the impacts of corn prices, the by-products of corn bioethanol production (i.e. distiller’s grains) are used as a high energy, high protein livestock feed supplement, which affects other components of livestock feed and forage intake [47] and potentially CH_4_ emissions from livestock consuming them [48].

## Discussion

### Evaluation of revised livestock CH4 emissions estimates

Compared to other bottom-up estimates for recent years (Table 5), our revised emissions factors yield annual CH_4_ C emission estimates that are: 11% larger than global estimates made using IPCC 2006 emissions factors; 15% larger than EPA global estimates but similar or slightly smaller than EPA US estimates; and 4% larger than EDGAR global estimates, 3% larger than EDGAR US estimates, but 54% larger than EDGAR estimates for the state of California. Our global estimates are slightly larger than those published for the 2000s by Tian et al. [49] based on a suite of bottom-up estimates, but have larger uncertainties. EDGAR uses IPCC 2006 Tier 2 calculations but modifies cattle emissions factors based on body weight or milk productivity; such modifications would not capture the effects of recent changes in manure management systems and other factors. EPA, in contrast, uses models with annually modified inputs for the US [50], but uses 2006 IPCC coefficients for its global estimates [16]. Our US emissions estimates are not significantly different from those made by EPA. This is not unexpected, as we use similar estimates for enteric fermentation emissions in US meat/other cattle, and rely on information from EPA to derive the new emissions factors for other livestock categories.

**Table 5:** Comparison of livestock CH_4_ C emissions reported in literature to revised values obtained in this study

Area	Years	Methane quantity	Source/emissions factors used	Method	Value (Tg C year^−1^)	Revised value (Tg C year^−1^)
Globe	2013	Total lvstk. CH_4_ C	IPCC 2006^a^	Bottom-up	81.3 ± 12.4	90.4 ± 13.8
			IPCC 2006 [36]		81.0	
		E.F. CH_4_ C	IPCC 2006^a^	Bottom-up	74.2 ± 11.4	80.6 ± 12.3
			IPCC 2006 [36]		73.8	
		M.M. CH_4_ C	IPCC 2006^a^	Bottom-up	7.14 ± 1.09	9.79 ± 1.5
			IPCC 2006 [36]		7.24	
Globe	2012	Total lvstk. CH_4_ C	IPCC 2006^a^	Bottom-up	80.9 ± 12.4	90.08 ± 13.78
			IPCC 2006 [36]		80.6	
		E.F. CH_4_ C	IPCC 2006^a^	Bottom-up	73.8 ± 11.3	80.3 ± 12.3
			IPCC 2006 [36]		73.4	
		M.M. CH_4_ C	IPCC 2006^a^	Bottom-up	7.10 ± 1.1	9.81 ± 1.5
			IPCC 2006 [36]		7.19	
Globe	2009–2011	Total lvstk. CH_4_ C	[20]	Top-down	88.94	87.88 ± 13.44 (2009)
						89.35 ± 13.67 (2011)
Globe	2010	E.F. CH_4_ C	[88]	Bottom-up	69.0	79.0 ± 12.1
		M.M. CH_4_ C			8.19	9.51 ± 1.5
Globe	2000s (average)	E.F. CH_4_ C	[49]	Bottom-up	70.0 ± 3.3	72.0 ± 11.0
		M.M. CH_4_ C			8.0 ± 0.3	8.4 ± 1.29
US	2012	E.F. CH_4_ C	IPCC 2006^a^	Bottom-up	4.38 ± 0.67	4.95 ± 0.76
			IPCC 2006 [36]		4.4	
			[89]		5.0	
		M.M. CH_4_ C	IPCC 2006^a^	Bottom-up	1.09 ± 0.17	1.93 ± 0.3
			IPCC 2006 [36]		1.02	
			[89]		1.91	
US	2009–2011	Total lvstk. CH_4_ C	[90]	Top-down	8.75–14.09	6.83 ± 1.05 (2009) 6.90 ± 1.06 (2011)
			[91]	Bottom-up	6.82 (2010); 6.77 (2011)	
			[12]		7.02 (2010); 6.97 (2011)	
			[89]		6.97 (2010); 6.91 (2011)	
California	2013–2014	Dairy cattle CH_4_ C	IPCC 2006^a^	Bottom-up	0.230 ± 0.035	0.382 ± 0.058 (2013)
			[51]	Top-down	0.603–1.06	
		All non-dairy lvstk. CH_4_ C	IPCC 2006^a^	Bottom-up	0.15 ± .022	0.168 ± 0.026 (2013)
			[51]	Top-down	0.149–0.259	
California	2010	Total lvstk. CH_4_ C	IPCC 2006^a^	Bottom-up	0.38	0.54 ± 0.08
			[5, as analyzed in 22]		0.35	
			[22]	Top-down	0.65	
US	2008	Total lvstk. CH_4_ C	[5, as analyzed in 23]	Bottom-up	6.7	6.88 ± 1.05
			[12]		7.09	
			[23]	Top-down	12.7 ± 0.5	
Globe	2008	E.F. CH_4_ C	[Sum over all gridcells from 5]	Bottom-up	75.12	77.82 ± 11.91
		M.M. CH_4_ C			8.65	9.14 ± 1.4
US	2004	Total lvstk. CH_4_ C	IPCC 2006^a^	Bottom-up	5.5	6.42 ± 0.98
			[92]		5.8	
			[21]	Top-down	9.15 ± 0.98	

*E.F.* enteric fermentation, *M.M.* manure management

^a^Our calculations, using IPCC 2006 [4] Tier 1 regional emissions factors

Our estimate of global livestock CH_4_ C emissions is similar to top-down estimates made using atmospheric inversion methods [20] (Table 5). Our estimates for the US, however, are smaller than recent top-down estimates by 21–51% [20], 46% [23], or 30% [21]. For the state of California only, our total-livestock estimate is 17% smaller than top-down [22] for 2010; for 2013, our estimate for non-dairy livestock was smaller but comparable, while our dairy cattle estimate was 37–64% smaller, than top-down [51] (Table 5). The differences over the entire US may be due in part to the difficulty in separating livestock CH_4_ emissions from other sources for the entire country in top-down studies [20]. US emissions could indeed be larger than our estimates, as suggested by these top-down studies; however, further investigation of this possibility will require more quantitative research on recent per animal emissions, particularly from increasingly used anaerobic manure treatment lagoons, such as recent studies on dairy cattle emissions by Owen and Silver [52, 53].

Our estimates for the state of California result in livestock emissions of 0.540 g CH_4_ C in 2010 and 0.550 Tg CH_4_ C in 2013, of which 0.165 and 0.177 Tg CH_4_ C are due to dairy cattle manure management, respectively. California, however, utilizes anaerobic lagoon manure management systems at a higher rate than the US national average (59% of manure is managed in anaerobic lagoons in California, compared to 34% for the US, based on state population-weighted values [50]). If we calculate emissions using the California manure management utilization rates in place of national average rates, California dairy cattle manure management emissions in 2010 and 2013 increase to 0.263 and 0.274 Tg CH_4_ C, bringing total California livestock emissions up to 0.638 and 0.647 Tg CH_4_ C for 2010 and 2013, respectively. These totals approach Wecht et al.’s value of 0.65 Tg CH_4_ C for 2010 [22], but are well below the range of 0.752–1.32 Tg CH_4_ C presented by Jeong et al. for 2013–2014 [51]. If we also employ MCF values from Owen and Silver’s recent field observations of anaerobic lagoon manure management systems [52], California dairy cattle manure management emissions in 2010 and 2013 increase to 0.306 and 0.318 Tg CH_4_ C, respectively, bringing total emissions increase to 0.681 and 0.691 Tg CH_4_ C in those years. These results show that our emissions estimates, if modified to reflect local conditions, are similar to or smaller than recent top-down estimates in California, where livestock and fossil fuel-sector CH_4_ emissions are spatially well separated. The discrepancies between top-down and bottom-up estimates may arise from factors influencing either or both of the methods. Our estimates could be too low for several reasons, including underreported usage of anaerobic manure treatment lagoons, recent increases in local temperatures impacting emissions, and/or MCF values that are too low. Because our emissions factors were calculated at the regional level, it will remain important to modify them when characterizing localized emissions; this can be done by using the equations published by the IPCC [4] with the inputs provided here in Tables 1 and 2, modified by relevant localized information such as manure management system utilization rates.

### Role of livestock CH_4_ in global atmospheric CH_4_ dynamics

In the early 2000s, annual increases in atmospheric CH_4_ concentrations temporarily flattened [24, 54]. After 2006, however, atmospheric CH_4_ concentration abruptly began to rise each year, and at the same time, its ^13^C isotopic signature began to grow more negative [26, 27]. Several possible explanations are offered for the causes and geographical distribution of this renewal in growth. Bergamaschi et al. [55] find that annual CH_4_ emissions (from all sources) in 2007–2010 were 16–20 Tg larger than emissions in 2003–2005 period, with the increase mostly in the Northern and Southern tropics and Northern mid-latitudes, and Nisbet et al. [25] indicate that global CH_4_ emissions (from all sources) were 15–22 Tg larger in 2010 than in 2005. Schaefer et al. [27] suggest that increases after 2007 are most likely from agricultural sources in the Northern hemisphere tropics and subtropics. In contrast, Nisbet et al. [26] suggest that these increases originate in the Southern hemisphere and Northern and Southern tropics, and are more likely due to wetland responses to meteorological conditions than agriculture, because of the abrupt step-change after 2006.

**Fig. 11:**
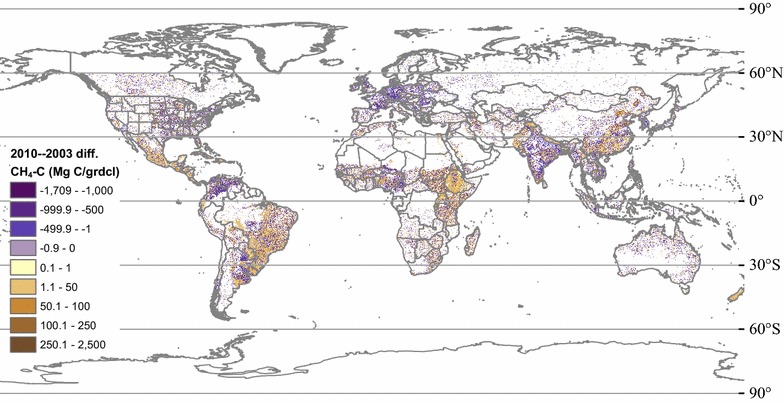
Change in total livestock CH_4_ emissions between 2003 and 2010 (2010−2003 emissions), per 0.05 × 0.05° gridcell

Our global estimates for annual livestock CH_4_ emissions are 118.0 Tg CH_4_ (88.5 Tg CH_4_ C) in 2010, 11.7 Tg CH_4_ greater than 2003 emissions of 106.3 Tg CH_4_ (79.7 Tg CH_4_ C). These quantities represent ca. one-fifth of total global methane emissions of 540–568 Tg CH_4_ year^−1^ estimated for this time period by a suite of top-down inversions [14]. The 11.7 Tg CH_4_ year^−1^ increase in annual livestock emissions reported here accounts for ca. one half to three-fourths of the increases over this time period reported by Bergamaschi et al. [55] and Nisbet et al. [25]. These proportions support the idea that livestock CH_4_ emissions, while not the dominant overall source of global CH_4_ emissions, may be a major contributor to the observed increases in emissions in the 2000s to 2010s. As suggested by Saunois et al. [28], the importance of agricultural emissions in the global CH_4_ budget is highlighted by our results, which provide quantitative estimates with associated uncertainties. It is important to note, however, that our results cannot reveal any sharp changes from year-to-year, because we have imposed a linear transition from IPCC-based to revised coefficients over the years from 1990 to 2012; therefore, a larger magnitude of change over this time period is possible. In summing the changes in annual livestock CH_4_ emissions over time by latitude (Fig. 11; Table 6), we find that the largest increases are between 30N and the equator (Northern tropics), potentially lending support to the conclusions of Schaefer et al. [27]. In the northern and southern tropics (30N to equator and equator to 30S), our results are comparable to the results of multiple models reported by Bergamaschi et al. [Table 3 in 55]; whereas in higher latitudinal zones, the changes over time that we document are the same in sign but smaller in magnitude than the output of most of the inversions reported by those authors. These longitudinal patterns may improve future discernment of CH_4_ sources and dynamics over time.

**Table 6:** Temporal changes in annual livestock CH_4_ emissions by latitudinal zone

Time period	90N–60N	60N–30N	30N-equator	Equator-30S	30S–60S	60S–90S
Tg CH_4_ C year^−1^						
2003–2010	−0.08 ± 0.15	1.7 ± 8.96	5.18 ± 9.87	2.01 ± 5.26	−0.16 ± 1.37	0
2000–2013	−0.12 ± 0.16	2.1 ± 8.97	5.59 ± 9.39	4.23 ± 5.19	0.28 ± 1.34	0
Tg CH_4_ year^−1^						
2003–2010	−0.11 ± 0.21	2.27 ± 11.95	6.91 ± 13.17	2.69 ± 7.02	−0.21 ± 1.83	0
2000–2013	−0.16 ± 0.21	2.8 ± 11.97	7.45 ± 12.51	5.64 ± 6.92	0.38 ± 1.78	0

Tg CH_4_ C and Tg CH_4_ both shown to facilitate comparisons to other studies

### Limitations of revised emissions factors

The revised per-animal emissions factors and/or total CH_4_ emissions reported here may differ from recent national self-reported emissions factors. This can be due to several factors, including (1) the inclusion of dairy calves and heifers with mature dairy cow populations, despite large differences in emissions between those groups, which can lead to low emissions factors; (2) interannual and sub-regional variation in diet and other factors. The revised emissions factors were developed for global analyses based on recent information, and the switch from IPCC 2006 was made linearly over a long time period (1990–2012) because information about their temporal dynamics was lacking. Therefore, variability at subregional and interannual scales are embedded in our estimates, and the revised emissions factors may not provide the best representation of emissions at local scales and/or for earlier years during the transition.

Emissions factors for poultry manure management CH_4_ were not revised in this study, but they should be reevaluated in future work. In the IPCC 2006 guidelines, poultry emissions factors for manure management are small (i.e. <0.10 kg CH_4_ per bird per year, except for laying hens with manure managed in lagoons). However, global poultry populations are large and continue to increase (Fig. 1b). Poultry manure management CH_4_ emissions are likely to be larger than estimated by IPCC 2006 guidelines for similar reasons as in cattle and swine: breeding has increased body sizes and growth rates [56] and utilization of liquid manure management systems is increasing [57].

The region of E. and SE Asia has proportionally less cattle and more swine than other regions of the world, making manure management of CH_4_ emissions much more prominent there (because swine do not have appreciable enteric fermentation emissions). The quantities of CH_4_ emitted from swine manure in this region, however, do have greater uncertainty. While the bulk of cattle manure in this region is collected and applied to cropland, particularly where cash crops are grown [9, 58–60], some of the manure produced by China’s large swine population (ca. half of the world’s total) is discharged to surface waters [61–64], and CH_4_ emissions have not been characterized for this situation. In this study, we considered emissions from manure discharged to surface waters to be similar to emissions from ‘liquid and deep pit’ treatments. If emission quantities from manure discharged to surface waters are more similar to emissions in anaerobic lagoons (i.e. liquid storage systems to combine waste stabilization and storage, in which solids are not removed more frequently than 15–20 years [65]), then the swine manure emissions factor may be as much as three times higher than the value used here.

In the Latin America region, meat cattle populations and their management practices are changing rapidly. In this region, our estimates of cattle on feedlots and other rapidly changing attributes may already be outdated [66], and frequent reassessment of this region will be warranted.

### US livestock CH_4_ emissions in recent decades

Total CH_4_ emissions for the US and Canada show a slight but steady increase over recent decades despite decreasing populations of dairy cows and other cattle. This contrasts with W. Europe, where total emissions trajectories decline slightly in parallel with declines in livestock populations. The means exist to further reduce CH_4_ emissions, and they are available in the US and Canada (e.g. covered manure storage). The centralization of manure management in this region increases profitability but is also associated with increasing CH_4_ emissions, decreased potential for cropland application of manure, and other threats to common resources and public health [7, 61, 67–69]. Studies examining the overall tradeoffs associated with increasing centralization vs. decentralization (e.g. potential C sequestration from manure application to cropland soils; NOx and CH_4_ emissions; costs of transportation for livestock, milk and meat, and manure; air and water quality; and impacts on rural communities such as odors and health risks) are needed. For example, if the efficiency of centralization outweighs other negative impacts, then capping lagoons to capture CH_4_ should be considered.

## Conclusions

In this study, we found the revised bottom up estimates of C fluxes and stocks from agricultural systems to be higher than those based on IPCC 2006 guidelines. The estimated global livestock CH_4_ emissions were 119.1 ± 18.2 Tg CH_4_ in year 2011; this quantity is 11% greater than that obtained using the IPCC 2006 emissions factors, encompassing an 8.4% increase in enteric fermentation CH_4_ and a 36.7% increase in manure management CH_4_, with notable variability among regions and sources. Likewise, the revised manure management CH_4_ emissions for year 2011 in the US were 71.8% higher than IPCC-based estimates, consistent with recently reported top-down estimates. Summing changes in annual livestock CH_4_ emissions geographically, by latitude and over time, we found that the largest increases over time were between 30N and the equator (i.e. Northern tropics). Our results suggest that livestock CH_4_ emissions, while not the dominant overall source of global CH_4_ emissions, may be a major contributor to the recent increases in global CH_4_ emissions. The new regional and global C fluxes and stocks estimates improve the ability to reconcile top-down and bottom-up estimates of CH_4_ production, and provide consistent estimates of CH_4_ emissions at the national, regional and global level, for use in development and evaluation of Earth system models and environmental assessments. The results reported here are useful to scientists and policy decision makers, given the importance of agricultural systems for food, fiber and bioenergy production, and their contributions to global methane emissions.
